# An Evidenced-Based Scale of Disease Severity following Human Challenge with Enteroxigenic *Escherichia coli*

**DOI:** 10.1371/journal.pone.0149358

**Published:** 2016-03-03

**Authors:** Chad K. Porter, Mark S. Riddle, Ashley N. Alcala, David A. Sack, Clayton Harro, Subhra Chakraborty, Ramiro L. Gutierrez, Stephen J. Savarino, Michael Darsley, Robin McKenzie, Barbara DeNearing, Hans Steinsland, David R. Tribble, A. Louis Bourgeois

**Affiliations:** 1 Enteric Disease Department, Infectious Disease Directorate, Naval Medical Research Center, Silver Spring, MD, United States of America; 2 Bloomberg School of Public Health, Johns Hopkins University, Baltimore, MD, United States of America; 3 MD Biologic, LTD, Cambridge, United Kingdom; 4 School of Medicine, Johns Hopkins University, Baltimore, MD, United States of America; 5 Centre for Intervention Science in Maternal and Child Health (CISMAC), Centre for International Health, and Department of Biomedicine, University of Bergen, Bergen, Norway; 6 Infectious Disease Clinical Research Program, Bethesda, MD, United States of America; 7 PATH, Washington, DC, United States of America; Instituto de Higiene e Medicina Tropical, PORTUGAL

## Abstract

**Background:**

Experimental human challenge models have played a major role in enhancing our understanding of infectious diseases. Primary outcomes have typically utilized overly simplistic outcomes that fail to entirely account for complex illness syndromes. We sought to characterize clinical outcomes associated with experimental infection with enterotoxigenic *Escherichia coli* (ETEC) and to develop a disease score.

**Methods:**

Data were obtained from prior controlled human ETEC infection studies. Correlation and univariate regression across sign and symptom severity was performed. A multiple correspondence analysis was conducted. A 3-parameter disease score with construct validity was developed in an iterative fashion, compared to standard outcome definitions and applied to prior vaccine challenge trials.

**Results:**

Data on 264 subjects receiving seven ETEC strains at doses from 1x10^5^ to 1x10^10^ cfu were used to construct a standardized dataset. The strongest observed correlation was between vomiting and nausea (r = 0.65); however, stool output was poorly correlated with subjective activity-impacting outcomes. Multiple correspondence analyses showed covariability in multiple signs and symptoms, with severity being the strongest factor corresponding across outcomes. The developed disease score performed well compared to standard outcome definitions and differentiated disease in vaccinated and unvaccinated subjects.

**Conclusion:**

Frequency and volumetric definitions of diarrhea severity poorly characterize ETEC disease. These data support a disease severity score accounting for stool output and other clinical signs and symptoms. Such a score could serve as the basis for better field trial outcomes and gives an additional outcome measure to help select future vaccines that warrant expanded testing in pivotal pre-licensure trials.

## Introduction

Dating back to variolation, the controlled human challenge model has played a major role in understanding disease pathogenesis, describing clinical and immunologic responses to infectious agents and testing the effectiveness of disease mitigating interventions (e.g. vaccines and prophylactics). Specifically, challenge models have been developed for vector-borne [1,2], respiratory [3,4] and gastrointestinal infections [5–7]. While these studies provide an opportunity to intently study a disease process in a well-controlled environment, their utility as a research tool for guiding the development of new prevention and control methods have frequently been met with several unique development challenges associated with variability in the challenge doses or strains used, which treatment protocol was implemented and how clinical outcomes were assessed [6].

In 1971 Dupont *et al*. published a seminal paper on the results of an experimental infection study with enterotoxigenic *Escherichia coli* (ETEC) which definitively identified this organism as a causative agent of acute infectious gastroenteritis [8]. Since that time, ETEC has become appreciated as a leading cause of diarrhea in children living in developing regions of the world and among adult travelers from developed areas to these same regions [9–11]. The epidemiologic burden associated with ETEC along with newly emerging markets for vaccine uptake has led to increased interest in the utilization of the controlled human ETEC infection to inform early vaccine development down-selection decisions. As the demand for these models increases, there is a need to improve our understanding of the value and utility which these models may provide and develop better outcome measures for discerning how interventions may impact on this spectrum of clinical illness.

To date, ETEC-attributable outcomes in experimental infections have been focused on defining diarrheal attack rates by counting and grading loose and liquid stools and calculating the proportion of subjects meeting *a priori* defined stool output definitions based on the frequency, volume and form of those stool specimens [6]. However, using only the frequency and volume of loose stools to define the severity of clinical illness resulting from experimental infection with ETEC fails to consider common associated symptoms and signs impacting subject well-being. To that end, we sought to describe the clinical outcomes associated with experimental ETEC infections and the overlap of those outcomes to facilitate future model development, refinement and utilization in the context of its anticipated future application to a rapidly expanding ETEC vaccine development program.

## Methods

Individual patient level data for this study were obtained from a series of previously published or presented experimental infection studies conducted by the US Department of Defense, Johns Hopkins Bloomberg School of Public Health, PATH Enteric Vaccine Initiative and by the University of Bergen/Haukeland University Hospital with a median year of conduct of 2006 [6,12,13]. To be included, subjects must not have received any investigational treatment or been previously infected with ETEC as part of an assessment of homologous protection prior to experimental infection with ETEC. These studies included the administration of 7 different ETEC strains (B7A, E24377A, H10407, LSN03-016011/A, WS0115A, DS26-1, TW10598) at doses from 1x10^5^ to 1x10^10^ colony forming units, and occurred over a 20 year period. The study protocol was approved by the Naval Medical Research Center Institutional Review Board in compliance with all applicable Federal regulations governing the protection of human subjects. Informed consent was not obtained for this study as all data were anonymized and de-identified prior to analysis.

In addition to data on the experimental challenge, strain, dose, time of antibiotic treatment relative to the time of receipt of the experimental infection, utilization of intravenous fluids, demographic information (age, gender, race/ethnicity) as well as detailed clinical information on the signs and symptoms associated with their illness were extracted and compiled into a single dataset. The following symptoms were documented as 0 –non-existent, 1 –mild (not interfering with routine activities), 2 –moderate (interfering but not precluding routine activities) or 3 –severe (precluding routine activities): malaise, abdominal cramps, headache and lightheadedness. Additionally, every episode of vomiting was documented. Fever severity was based on maximum measured temperature as follows: no fever (<38.0°C), mild (38.0–38.4°C), moderate (38.5–38.9°C), severe (≥39.0°C); while vomiting severity was coded as: no vomiting (0 episodes), mild (1 episodes), moderate (2 episodes), or severe (≥3 episodes). Symptom severity was based on the maximum observed severity during the course of the infection. While diarrhea was originally defined by each study independently, for standardization we re-defined diarrhea severity based on the following definition: mild (1 loose/liquid stool of ≥300 g or ≥2 loose/liquid stools totaling ≥200 g and < = 400 g during in a 24 hour period), moderate (4 to 5 loose/liquid stools or >401 to 800 g in a 24 hour period), severe (6 or more loose/liquid stools or ≥800 g in a 24 hour period). The total stool amount of loose/liquid stools (frequency and weight) as well as the maximum number in a 24 hour period, the time to diarrhea onset and the duration of diarrhea relative to experimental infection was also included in the database. Stool output was re-analyzed based on the distribution of stool frequency and volume (1g = 1 ml).

The prevalence and severity of each of the signs and symptoms were reported based on the original observed findings and the application of the definitions described herein. Spearman correlations of ordinal severity of signs and symptoms were estimated using a Fisher’s Z transformation [14,15]. Univariate, linear regression was utilized to describe the strength of the association between stool output and other ETEC-attributable signs and symptoms. The distribution of stool output was assessed and ordinal groups of stool output developed based on median and the interquartile ranges of output. A multiple correspondence analysis was also performed to describe the overlap of the severity of all signs and symptoms. Briefly, a multiple correspondence analysis is a method by which the relationship between numerous nominal and/or ordinal data can be described graphically [16]. First the data were converted to a Burt table, or indicator matrix, effectively a *K* by *K* table of all possible pairwise tabulations of the categorical data. When graphically displayed, the proximity of points, on a two dimensional graph, represents the relationship between those variables among the observed data (i.e., the more proximal variables in a two-dimensional plane, the more similar their distribution).

Results from the multiple correspondence analysis were utilized to identify groups of corresponding clinical outcomes for compilation into a disease score. The disease score was iteratively developed with goals of parsimony and normality, and assessed using a receiver-operator curve (ROC) based on its ability to predict the traditional primary outcome of moderate to severe diarrhea. The *a priori* constructed model was applied to previously performed and published vaccination/challenge trials for which individual line listing data were available [17–19]. Differences in the disease scores between groups were compared using a Student’s t-test with a 2-sided *alpha* = 0.05.

## Results

Data were obtained on 264 subjects as outlined in Table 1. Subjects were predominately male (65.2%) and African-American (68.9%) with a median age of 31.3 (interquartile range {IQR}: 24.0, 41.0). There were slight, but statistically significant differences in the distribution of races across the studied strains (p<0.01), participant age (p<0.01) and gender (p = 0.02).

**Table 1:** Demographics of study population.

Strain	H10407	E24377A	B7A	LSN03*	WS0115A	DS26-1	TW10598
**CFs**	CFA/I	CS1, CS3	CS6, CS21	CS17	CS19	CS19	CS2, CS3, CS21
**Toxins**	LT, ST	LT, ST	LT, ST	LT	LT, ST	LT	LT,ST
**Doses (cfu)**	10^5^−10^9^	10^8^−10^9^	10^9^−10^10^	10^8^−10^9^	10^8^−10^10^	10^8^	10^6^−10^9^
**N**	132	36	16	25	20	5	30
**Median age**	33.5	38.0	40.7	32.9	34.3	28.7	23.0
**% Male**	67.4	80.6	68.8	64.0	75.0	60.0	30.0
**% Af.Am.**	76.5	77.8	43.8	88.0	95.0	100	0.0
**References**	[12,17,30–32]	[33,34]	[30]	[35,36]	[35]	[35]	[13]

* Complete strain name: LSN03-016011/A

cfu, colony forming units; IQR, interquartile range; AA, African-American; CFs, colonization factors; LT, heat-labile toxin; ST-heat-stable toxin.

As shown in Table 2, subjective symptoms were observed in a majority of subjects with abdominal cramps and malaise most commonly observed (62.9 and 47.0%, respectively). In contrast, the objective signs and symptoms of fever and vomiting were less common (15.2% and 20.1%, respectively). The outcome of diarrhea was observed in 71.6% of challenged subjects with 48.7% of those characterized as ‘severe’ diarrhea based on traditional volume and frequency criteria.

**Table 2:** Frequency (%) of signs and symptoms.

	Diarrhea	Cramps	Headache	Lightheaded	Malaise	Nausea	Vomiting	Fever
**None**	28.4	37.1	59.1	74.1	53.0	54.5	79.9	84.8
**Mild**	13.3	20.5	21.2	12.2	15.9	18.2	6.1	12.5
**Moderate**	23.5	24.6	12.1	10.6	15.9	8.7	3.0	2.3
**Severe**	34.9	17.8	7.6	3.0	15.2	18.6	11.0	0.4

There was a statistically significant correlation between all analyzed signs and symptoms of ETEC-attributable illness; however, the strength of correlation varied. The strongest correlation observed was between vomiting and nausea (ρ = 0.65); however, the number of subjects that experienced at least a single episode of vomiting was relatively low (20.1%). Nausea and vomiting were both strongly correlated with malaise (ρ = 0.56 and 0.50, respectively, Table 3) while malaise and nausea were correlated with abdominal cramps (ρ = 0.57 and 0.60, respectively). More objective outcomes such as diarrhea, vomiting and fever were less strongly correlated. In particular, the strongest correlation between diarrhea severity and any other signs and symptoms was for abdominal cramps and malaise (at ρ = 0.45 and 0.44, respectively).

**Table 3:** Spearman correlations of ordinal signs and symptoms of ETEC illness.

Sign/Symptoms		Correlation estimate (ρ)	Correlation estimate (ρ) 95% confidence limits		p-value
			Lower	Upper	
**Diarrhea**	Abdominal cramps	0.45	0.35	0.54	<0.001
	Headache	0.18	0.06	0.29	0.004
	Lightheadedness	0.32	0.21	0.43	<0.001
	Malaise	0.44	0.33	0.53	<0.001
	Nausea	0.41	0.30	0.50	<0.001
	Vomiting	0.33	0.22	0.44	<0.001
	Fever	0.28	0.17	0.39	<0.001
**Abdominal cramps**	Headache	0.35	0.24	0.45	<0.001
	Lightheadedness	0.38	0.27	0.48	<0.001
	Malaise	0.57	0.48	0.64	<0.001
	Nausea	0.60	0.51	0.67	<0.001
	Vomiting	0.46	0.35	0.55	<0.001
	Fever	0.16	0.04	0.27	0.011
**Headache**	Lightheadedness	0.31	0.20	0.42	<0.001
	Malaise	0.33	0.21	0.43	<0.001
	Nausea	0.31	0.20	0.42	<0.001
	Vomiting	0.18	0.06	0.30	0.003
	Fever	0.14	0.02	0.25	0.025
**Lightheaded**	Malaise	0.54	0.45	0.62	<0.001
	Nausea	0.41	0.30	0.50	<0.001
	Vomiting	0.44	0.33	0.53	<0.001
	Fever	0.28	0.16	0.38	<0.001
**Malaise**	Nausea	0.56	0.47	0.63	<0.001
	Vomiting	0.50	0.40	0.58	<0.001
	Fever	0.27	0.16	0.38	<0.001
**Nausea**	Vomiting	0.65	0.57	0.71	<0.001
	Fever	0.32	0.21	0.42	<0.001
**Vomiting**	Fever	0.33	0.21	0.43	<0.001

Note: Ordinal values of signs and symptoms: 0, none; 1, mild; 2, moderate; 3, severe).

**Fig 1:**
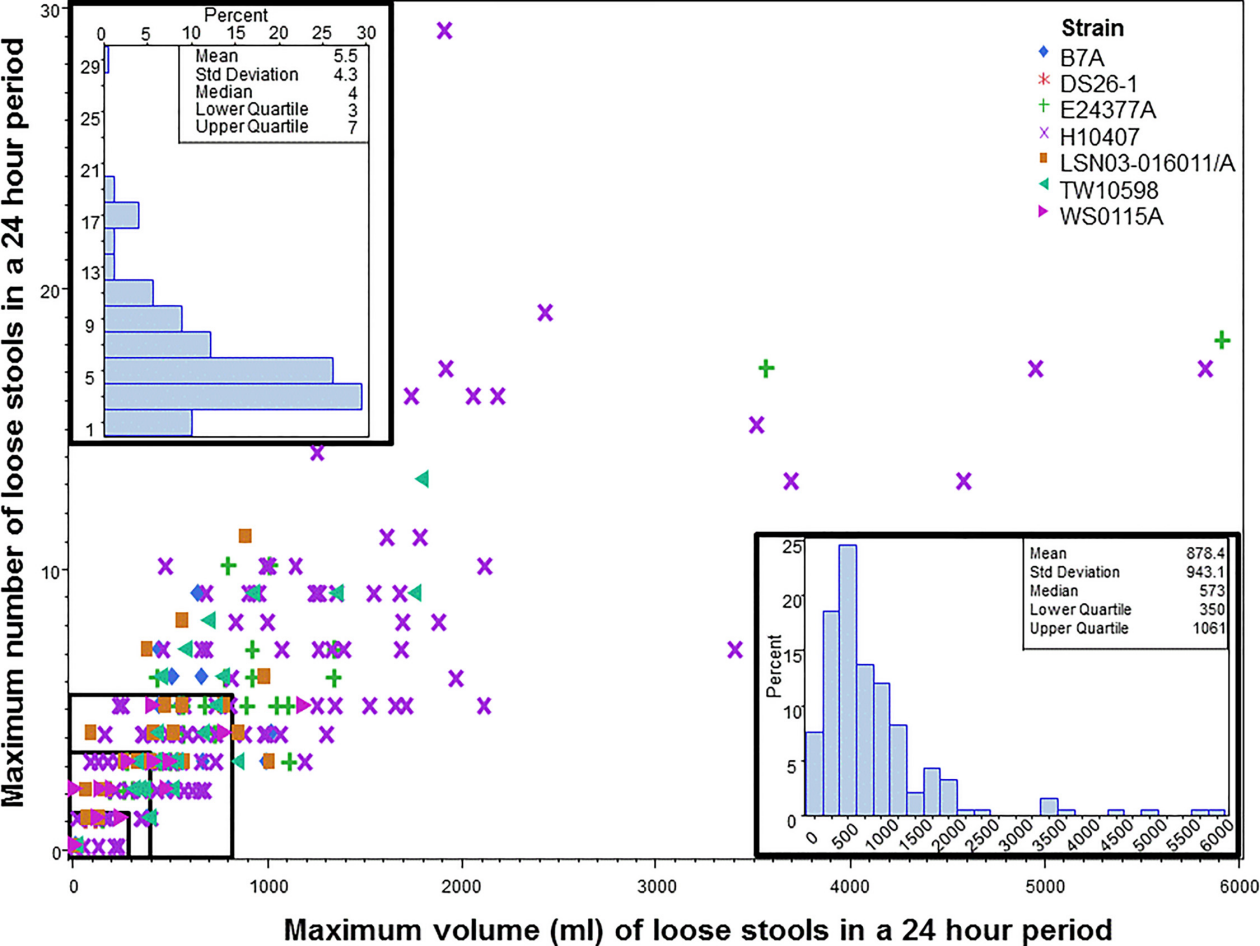
Maximum number and volume of loose stools during any 24 hour period post-inoculation. Footnote: Rectangles represent thresholds for mild, moderate and severe diarrhea.

The maximum stool output (frequency and volume) in any 24 hour period among those with any loose stools following challenge is shown in Fig 1. Neither the frequency nor the volume of stool was normally distributed with the highest proportion of subjects producing <6 episodes and <1 L in any 24 hour period. Specifically, during the peak 24 hour output period, the median number of stools produced was 4 (IQR: 3, 7). Similarly, the median stool volume during the peak 24 hour output period was slightly more than 500 milliliters (ml) (IQR: 370 ml, 1013 ml). There was a strong, statistically significant correlation (*r* = 0.80; p<0.001) between stool frequency and volume among subjects with less than 2 liters (L) and fewer than 10 loose stools in a 24 hour period. However, that correlation was less apparent among individuals with a higher frequency or volume of output (*r* = 0.34, p = 0.08). The rectangles in the bottom left corner of Fig 1 highlight increasing diarrhea severity from ‘none’ to ‘severe’ based on traditional cut-points for stool frequency and volume. While the output for mild and moderate diarrhea is tightly grouped, the output associated with severe diarrhea represents a much more broad distribution of stool frequency and volume.

Univariate regression models (Table 4) showed statistically significant associations between each of the signs/symptoms excluding headache and the maximum 24 hour stool volume. Variability in the severity of non-stool output signs and symptoms accounted for 0.4% to 23% of the variability in the maximum amount of stool produced in a 24 hour period. Similar association was seen with stool frequency with up to 24% of the variability described by other signs and symptoms. Multivariate regression models were not possible due to the high degree of multicollinearity across variables.

**Table 4:** Univariate regression between ETEC-attributable signs and symptoms and maximum 24 hour stool output.

Sign/Symptom	Maximum 24 stool volume			Maximum 24 stool frequency		
	Adjusted R^2^	β	p-value	Adjusted R^2^	β	p-value
Abdominal cramps	0.1236	274.3	<0.001	0.1541	1.5	<0.001
Headache	0.0038	79.1	0.16	0.0313	0.8	0.002
Lightheadedness	0.1147	374.1	<0.001	0.1181	1.9	<0.001
Malaise	0.2258	366.2	<0.001	0.2420	1.9	<0.001
Nausea	0.2320	360.6	<0.001	0.2421	1.8	<0.001
Vomiting	0.1816	380.1	<0.001	0.1855	1.9	<0.001
Fever	0.1223	662.1	<0.001	0.1122	3.2	<0.001

**Fig 2:**
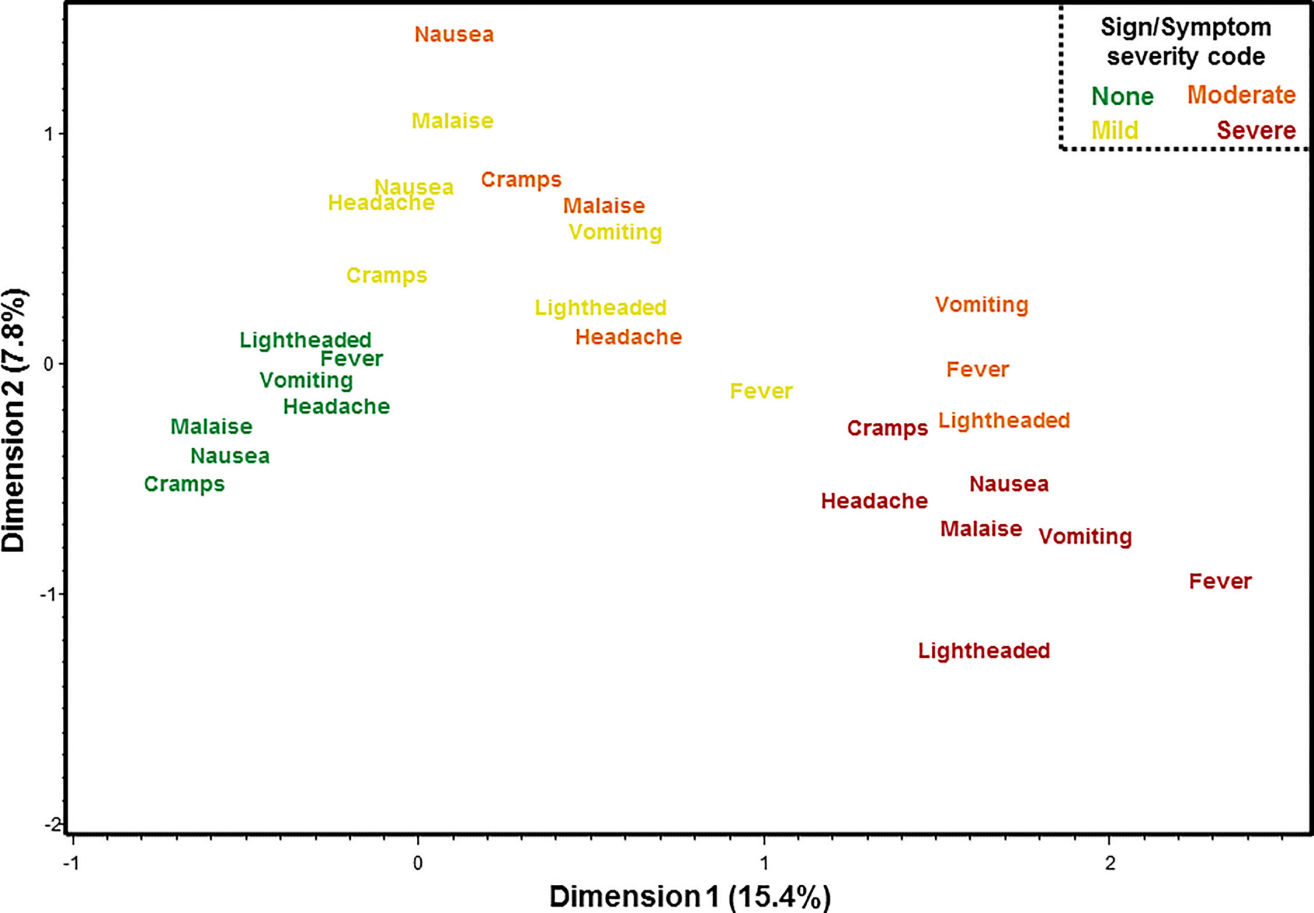
Multiple correspondence analysis of signs and symptoms of ETEC following experimental infection.

Multiple correspondence analyses (MCA) showed covariability in multiple signs and symptoms with severity being the most common factor associated with similar dimensions in two-dimensional space (Fig 2); although the two dimensions only accounted for just over 33% of all the variability. Specifically, the lack of any symptoms and no diarrhea or fever grouped tightly together. Similarly, severe signs and symptoms other than diarrhea were also tightly grouped. Mild signs and symptoms were mostly grouped in the upper left hand quadrant; however, mild fever tended to group with other severe signs and symptoms. Interestingly, all degrees of fever severity had comparable coordinates on Dimension 1 and were relatively comparable on Dimension 2. Moderate signs/symptoms appeared more inter-dispersed with some (nausea, abdominal cramps) appearing with other mild signs/symptoms, others (lightheadedness) more commonly associated with other severe signs/symptoms and still others (vomiting, fever) appearing to not have a high degree of correspondence. Severe diarrhea appeared most proximal to mild and moderate symptoms.

**Fig 3:**
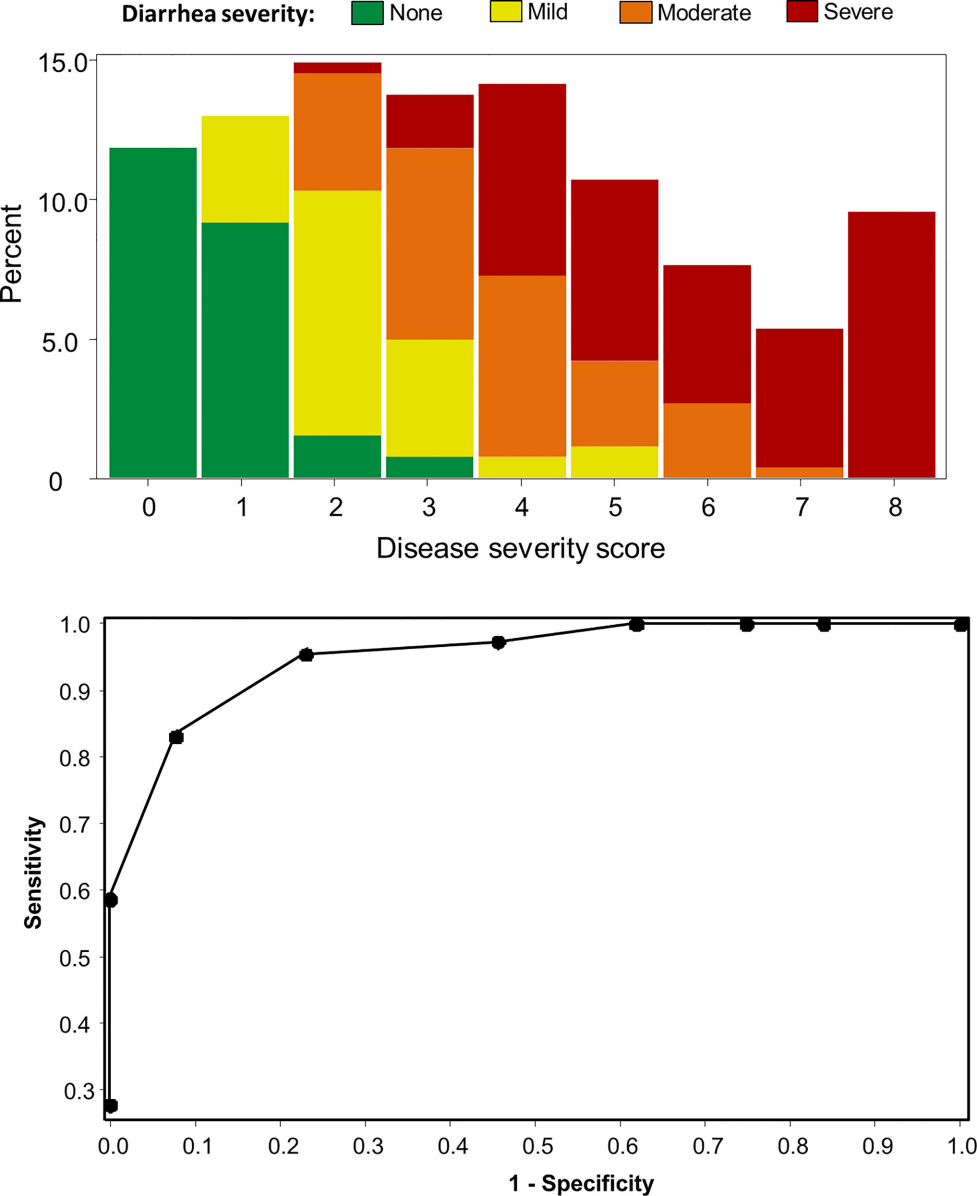
A: Histogram of 3 component ETEC disease score relative to traditional diarrhea severity based on fixed cut points of stool output. B: Receiver Operator Curve comparing 3 component ETEC disease score to traditional primary endpoint of moderate-severe diarrhea based on stool output.

Based on the grouping of clinical outcomes in the MCA and distributions of stool output with the goals of parsimony, normality, and ROC optimization, a three-component disease score was developed utilizing the objective signs, subjective symptoms and stool output (Table 5) yielding a score ranging from 0 (no disease) to 8 (most severe disease). The frequency distribution of disease score for 264 subjects included are shown in Fig 3A and overlaid with the more traditional diarrhea severity output based solely on loose stool output. Intuitively, with an increasing disease score, the proportion of subjects characterized as severe increases; however, there is some variability with subjects with relatively lower disease scores who have met the traditional severe diarrhea definition. This is further highlighted in the ROC shown in Fig 3B. Specifically, as the disease score increases, sensitivity approaches 100% with a high degree of sensitivity and specificity with disease scores of 2 to 3.

**Table 5:** Disease severity score components.

Parameter	Outcome		Score
**Objective signs**	>1 episode of vomiting/24 hrs ***OR*** any fever		2
	1 episode of vomiting ***AND*** no fever		1
	No vomiting ***AND*** no fever		0
**Subjective symptoms**	Moderate-severe lightheadedness ***OR***		2
	Severe: nausea, malaise, headache or abd cramps		2
	Mild lightheadedness ***OR***		1
	mild-mod: nausea, malaise, headache or abd cramps		1
	No ‘subjective symptoms’		0
**Diarrhea score (max 24 hr loose stools)**	>1000 ml	>12 episodes	4
	>600 to ≤1000 ml	>7 to 12 episodes	3
	>400 to ≤600 ml	>4 to ≤7 episodes	2
	>0 to ≤400 ml	1 to 4 episodes	1
	No loose stools	No loose stools	0

Footnote: diarrhea score assigned by the highest score determined by either maximum 24 hour output volume or frequency.

Application of the disease score to previously conducted vaccination/challenge trials was subsequently used to evaluate the potential utility in intervention discrimination (Table 6). Specifically, a study performed by McKenzie *et al* utilizing the CS1/CS3 LT+ST+ ETEC strain E24377A to assess preliminary efficacy of the LT patch vaccine yielded no significant differences (p-value = 0.35) in the mean disease scores in the vaccine (3.68) and placebo (4.33) groups, consistent with the per protocol analysis demonstrating no significant efficacy [18]. Of note, the subsequent phase 3 trial with the LT patch failed to demonstrate efficacy against LT+ST+ producing ETEC [20,21]. When the disease score was applied to a recent trial assessing the efficacy of the live-attenuated ETEC vaccine, ACE527 (TD Vaccines and PATH sponsored study), against H01407 challenge, a discrimination in efficacy was more readily seen compared to the original *a priori* analyses. In the first study, in which 2 doses of ACE527 or placebo were administered 3 weeks apart, there was a borderline significant difference (p = 0.07) in mean disease score between placebo (4.69) and vaccine (3.31) recipients (29% reduction) [17]. The primary analysis for this study focusing on moderate-severe diarrhea yielded an efficacy estimate of 26.5% (1-sided p-value: 0.12); however, upon post-hoc analyses, the researchers noted a significant effect on other secondary outcomes. A follow-on trial with an improved ACE527 construct which assessed a 3-dose series (days 0, 28, 56) as well as an adjuvant (LTR192GL211A) demonstrated almost a 50% reduction in the mean disease score between the adjuvant vaccine group and unvaccinated subjects (p = 0.03), again, more discriminative than the primary analyses focused on stool outcomes alone [22].

**Table 6:** Comparison of 3-component ETEC disease score in naïve and vaccinated subjects.

Author (Reference)	Challenge strain	Group	N	Mean (std) Score	2-sided p-value
McKenzie^1^ [18]	E24377A	Placebo	15	4.33 (2.23)	—
		LT Patch	19	3.68 (1.80)	0.35
Darsley [17]	H10407	Placebo	26	4.69 (2.48)	—
		ACE 527	29	3.31 (3.05)	0.07
Darsley [19] ^2^	H10407	Naïve/placebo	31	4.35 (2.70)	—
		ACE 527	13	3.92 (2.93)	0.64
		ACE 527 + dmLT	13	2.38 (2.60)	0.03

^1^ Limited to a subset of subjects based on available data

^2^ No significant differences in the mean scores for subjects receiving placebo (4.40) and naïve (4.33) subjects challenged concurrently (p = 0.95).

## Discussion

As outlined in Table 2, experimental infection studies with ETEC to date have characterized 50% of the diarrhea cases as ‘severe’ and only 18% as ‘mild’. This asymmetrical distribution may reduce the discriminatory potential of interventions leading to type II error, with potential negative implications for vaccine development given that these experimental infection models are often utilized as an early assessment of vaccine efficacy. As shown here, a large proportion (50%) of subjects with ‘any’ diarrhea met the severe definition, yielding a severity profile that may not be commensurate with natural infection. For example, Matilla *et al* reported a median of 5.5 unformed stools among travelers with ETEC-attributable illness during the first 24 hours of illness [23]. Similarly, among travelers to Turkey, only about 35% of those with ETEC-attributable travelers’ diarrhea had ≥6 loose stools in the 24 hour period prior to seeking medical care [24]. In direct contrast, however, 43% of travelers to Mexico and Guatemala with ETEC-attributable diarrhea met a comparable definition for severe diarrhea (≥6 loose stools in a 24 hour period) [25]. One potential option to mitigate unequal distribution is to establish new frequency/volume cut-points, such as the median and interquartile ranges, based on the data observed to date. These modified outcomes would ensure a more equal distribution of diarrhea cases across the spectrum of severity. However, it should be noted that included studies are unequally distributed among ETEC strains. Specifically, subjects receiving H10407 account for 50% of all analyzed subjects and this strain is noted to have a higher rate of stool output and more severe associated signs and symptoms [6]. The establishment of new stool output cut-points or other disease-defining characteristics should carefully consider the differential representation of a strain associated with more severe disease.

In addition to modifying the stool output cut-points, there is an increasing need to consider non-stool related objective and subjective signs and symptoms that are an important part of the diarrheal disease syndrome. As noted in Table 3, there is a relatively weak (though statistically significant) correlation between the severity of numerous signs and symptoms and stool output, defined as diarrhea severity. Failing to incorporate these signs and symptoms as part of the overall disease process is problematic as these complaints often dictate the impact of the disease on a subject’s ability to perform his/her normal activities. For example, among the 110 subjects analyzed here with mild or no diarrhea based on stool outcomes, many had moderate-severe symptoms not directly related to stool output such as abdominal cramps (25.5%), malaise (18.2%), headaches (13.6%) and nausea (10.9%). Furthermore, in a study or healthcare seeking behavior for acute TD by Sanders *et al*., diarrhea frequency and duration were not discriminating factors, but vomiting and fever were significant predictors (John W. Sanders, submitted). Arguably these signs and symptoms, which, by definition interfere with or preclude one’s normal activities, represent an important, yet currently unmeasured, characteristic of the overall diarrhea severity classification. In studies of travelers, a general functional assessment is often included to assess for the impact of illness on one’s ability to perform normal activities. Sanders et al reported that 23% of subjects with ETEC-attributable TD were unable to perform their normal activities [26]. The compilation of signs and symptoms that precluded activities is unclear; however, it is reasonable to assume it included stooling patterns in addition to other gastrointestinal and systemic signs and symptoms.

Furthermore, developing a disease scoring algorithm that incorporated outcomes other than stool output could have great application in the field. In the absence of the ability to quantify stool volume in the field, vaccine efficacy studies have frequently relied solely on self-reported number of loose stools. Most recently, two separate field studies of the efficacy of an LT skin patch vaccine were conducted [20,21]. The primary outcome of those studies was ETEC-attributable moderate-severe diarrhea defined as ≥4 loose stools in a 24 hour period. While tertiary endpoints included impact of illness on daily activities, those outcomes were not factored into the observed illness to assess disease severity. The classical “travelers’ diarrhea” definition does incorporate other symptoms (often fever, abdominal cramps, tenesmus, nausea, vomiting, and passage of bloody stools) [11]; however, this definition does little to differentiate illness severity, of great importance in vaccine efficacy studies for diarrheal illness where focus in on prevention of moderate to severe disease. The inability to quantify stool volume in this setting, and by necessity rely on stool frequency, may further obfuscate disease severity. Specifically, stooling habits during acute illness are likely impacted by baseline stool habits, dietary habits during travel and illness, tenesmus or pre-tenesmus types of sensations and/or other factors that may modulate stooling patterns and result in disease classification that may not be commensurate with the overall individual impact of the syndrome.

Herein, we have proposed a disease score for application to prior, current and future human challenge models with ETEC. In addition to overcoming many of the limitations of traditional outcome measures outlined above, application of a disease score may increase statistical efficiency. Specifically, studies have to date incorporated multiple pair-wise comparisons of various signs and symptoms without alpha adjustments increasing the likelihood of making a Type II error. This can be minimized by comparing a multicomponent disease score that compiles these parameters into a single measure. Furthermore, a dimensional score that can be subjected to parametric statistical methodologies, may have more discriminating power. Similar disease scores have been adopted and applied for rotavirus and norovirus-attributable illnesses; however, their direct application in the setting of ETEC experimental human challenge is limited by the parameters utilized [27,28]. For example, application of the modified Vesikari score described by Atmar *et al* utilizes diarrhea duration, a parameter that is highly influenced by the use of antibiotics in the setting of ETEC challenges [28]. Additionally, parameters on the duration and severity of vomiting may be less important indicators of ETEC-attributable illness severity. Tribble et al. attempted to develop a disease severity classification for human challenge studies with *C*. *jejuni* [29]; however, hypothesis-free approaches such as the one utilized here may be less susceptible to bias. Nonetheless, the success of these scores in early clinical assessment of prototype vaccines supports their development and use for non-viral etiology agents of enteric disease.

As with all models, there are inherent limitations with the disease score proposed herein. Specifically, this study is limited in its ability to predict a meaningful outcome. While we have shown the ROCs associated with the disease scores ability to predict traditionally defined moderate-severe diarrhea, we have argued that moderate-severe diarrhea as currently measured by stool output is a sub-optimal outcome in these controlled human infection studies. One opportunity to refine this may be the consistent collection of validated measure of a subject’s ability to perform his/her planned activities (travel, perform normal functions, etc) and assess the ability of the proposed disease score in predicting decreasing activity. Additionally, while the outcomes are harmonized as much as possible, there is potential variability in how the more subjective measures are reported and recorded across subjects and/or investigators and future studies should ensure collection of these parameters with focus on harmonization with existing methods. Furthermore, the outcomes included in this score are not the only possible measures that can be obtained in controlled human infection models and consideration should be given to these additional measures in future studies. Also, host-specific parameters not currently included in these analyses, such as diet and microbiome, may impact disease outcomes. In an effort to harmonize the data, it may be that the characterization of new stool output measures may have also impacted the influence of the non-stool related outcome measures.

Unequal distribution of diarrhea severity may be problematic given the use of these models to assess vaccine efficacy with primary outcomes targeting the prevention of moderate-severe disease. Additionally, failing to incorporate other signs and symptoms of disease is problematic as it is often these additional complaints that influence a disease’s functional impact. These data support the development of a disease severity scoring algorithm that accounts for stool output and also includes other important clinical signs and symptoms. In addition, the development of such a scoring algorithm could have great utility in field trials where studies necessarily rely on self-reported outcomes.
